# Special Issue “Recent Progress in Regenerative Therapy Using Blood-Derived Biomaterials”

**DOI:** 10.3390/ijms27073095

**Published:** 2026-03-28

**Authors:** Tomoyuki Kawase, Takashi Ushiki, Tomoharu Mochizuki

**Affiliations:** 1 Division of Oral Bioengineering, Institute of Medicine and Dentistry, Niigata University, Niigata 951-8514, Japan; 2 Department of Transfusion Medicine, Cell Therapy and Regenerative Medicine, Niigata University Medical and Dental Hospital, Niigata 951-8520, Japan; tushiki@med.niigata-u.ac.jp; 3 Department of Orthopaedic Surgery, Graduate School of Medical and Dental Sciences, Niigata University, Niigata 951-8510, Japan; 4 Division of Hematology and Oncology, Graduate School of Health Sciences, Niigata University, Niigata 951-9518, Japan; 5 Department of Hematology, Endocrinology and Metabolism, Faculty of Medicine, Niigata University, Niigata 951-8510, Japan

What are the primary problems with current platelet-rich plasma (PRP) therapy? The answer may vary across medical fields; however, a common problem is the lack of evidence [1,2,3,4]. PRP efficacy has been well established in preclinical studies, including in vitro experiments and animal models. In clinical studies, PRP has been shown to be effective through appropriate case selection. However, there is a lack of clear evidence from randomized clinical trials involving multiple facilities [5,6,7]. Real-world clinical outcomes are sometimes disappointing or questionable in specific medical fields [8,9,10]. In interpreting this discrepancy, it is most likely that the efficacy of PRP is affected by unidentified factor(s) in older adults, even though it remains sufficiently effective under “ideal” human conditions.

To address this issue, or to at least offer guidance toward a resolution, it is more important to deepen our understanding than to accumulate clinical data without sufficient quality control or to meta-analyze those confusing data. This Special Issue aims to collect research on novel technologies, insights, perspectives, and preclinical and clinical studies across medical fields on PRP derivatives.

More than 10 articles were submitted, and nine papers were accepted for publication. In their review article, Chalidis et al. (Contribution 1) examined the literature on the efficacy and safety of PRP in patients with patellofemoral osteoarthritis (OA), chondromalacia patellae, and anterior knee pain. These authors emphasized the need for well-designed, appropriately powered randomized trials to validate the efficacy of PRP, as has been the case in many other medical fields. The authors also clearly stated the need to establish patient selection criteria. To avoid unsuccessful therapy, it is generally thought that clinicians should identify non-responders before treatment. However, specific technologies to meet these clinical needs have not yet been developed. Therefore, we hope that this review will inform improvements in current PRP therapy, particularly its predictability.

Pasternak et al. (Contribution 2) introduced intra-articular injections of autologous conditioned serum (ACS) to treat temporomandibular joint disorder and reviewed its clinical potential. Although ACS was previously reviewed for the treatment of musculoskeletal diseases, particularly OA [11], this therapeutic option is not widely accepted or approved among many oral surgeons. However, this idea is not new and has long been used as an innovative therapy by oral surgeons. We hope this article will pave the way for developing a prototype of this therapy in the near future.

In another review article, Stępień et al. (Contribution 3) reviewed the usefulness of blood derivatives in treating ocular surface diseases. PRP eye drops are considered safe and effective for the treatment of dry eye disease and are used in many clinics [12]. In this sense, this topic is not new. However, to the best of our knowledge, few studies have reported non-responders or unsuccessful clinical outcomes. Thus, it may be beneficial for PRP clinicians and patients to consider the specific mechanism of action of PRP alongside the pathophysiological specificity of the ocular surface. It will also be helpful to discover the “disturbing” factors described in the initial paragraph.

In the standardized administration protocol, liquid PRP should be activated to form a gel to prevent immediate enzymatic degradation and passive diffusion. Platelet-rich fibrin (PRF), a gel-like matrix often referred to as the “2nd generation of PRP,” can be prepared without activators (i.e., coagulation factors). This soft matrix contains growth factors similar to those in liquid PRP preparations [13] and retains them for time-dependent release in vitro [14]. Dohle et al. (Contribution 4) conducted a proof-of-concept study to demonstrate the controlled release capacity and periodontal cell proliferation of PRF in a periodontal tissue model by precisely combining PRF with cells from the same donor. This study may attract the attention of clinicians who have been dissatisfied with the low reproducibility of PRP/PRF therapy.

Sánchez and his co-workers focused on the extracellular plasma fraction and its usefulness (Contribution 5). This concept is be based on autologous protein solution (APS) [15], which has already been implemented with the launch of a specific preparation kit, the nSTRIDE APS Kit (Zimmer Biomet) [16]. These authors developed a new formulation by removing leukocytes and their inflammatory cytokines from APS and proposed it as a better treatment option for OA. They also provided new insights for PRP users in their original research (Contribution 6). Many clinicians believe that PRP efficacy is primarily determined by platelet count rather than platelet quality or extracellular plasma quality [17,18]. These authors challenged this stereotype by demonstrating that increasing the plasma concentration improves fibrin-scaffold formation, potentially enabling more efficient controlled release of growth factors.

There is no doubt that antioxidants are the universal key to protecting our blood from the relentless attack of reactive oxygen species (ROS), which induce and exacerbate inflammation depending on their local levels [19,20]. Although the efficacy varies among individuals, plasma is rich in ROS scavengers [21]. We also confirmed that plasma has a higher antioxidant capacity and maintains this potential even after a brief heating step to convert liquid platelet-poor plasma into a gel [21,22,23,24]. Therefore, PRP has long been considered to function as a strong antioxidant [25], which is one of the reasons for its pain control capacity. Tognoloni et al. (Contribution 7) were the first to reveal the mechanism of action at the molecular level. However, plasma antioxidant capacity does not change significantly with age, sex, or physique; rather, it varies primarily with diet [26]. At present, many researchers and clinicians do not recognize the need for health promotion to respond more sensitively to PRP treatment. This study sheds light on the fundamental aspects of the other side of PRP therapy.

Based on clinical experience shared with sports doctors in the surrounding area, Mochizuki et al. aimed to investigate the potentially greater efficacy of PRP therapy in athletes. Despite the hypothesis that growth factor levels are significantly elevated in athletes, these authors found no substantial increases in growth factor levels in their two original research papers (Contributions 8 and 9). Instead, they demonstrated that PRP has a greater anti-inflammatory capacity in athletes. This finding suggests that the capacity to control inflammatory conditions may be greater among athletes than among sedentary adults. In addition, it has been suggested that, similar to PRP quality, the recipient’s physical condition can significantly influence PRP efficacy [27]. Elite athletes, particularly professional athletes, suffer from constant, excessive physical and mental stress that suppresses their self-defensive immune systems. Increased anti-inflammatory factors may be a compensatory mechanism for immunosuppression. These studies have paved the way for improving the efficacy of PRP therapy in athletes.

All studies published in this Special Issue are expected to contribute to the progress of PRP research and therapy. However, we should recognize that current forms of PRP therapy have unavoidable and unsolvable limitations, particularly in discerning inapplicable cases and non-responders [4,28,29]. The success of PRP therapy is influenced by PRP quality and patient conditions [30]. Owing to their safety and cost-effectiveness, autologous PRP preparations can be readily introduced in individual clinics without high initial costs [31]. The major limitation of this preparation option is that it does not ensure PRP quality for regenerative therapy, although it is sufficiently effective for pain control. Currently, nothing can be done to compensate for the age-related decline in PRP quality. Allogeneic PRP has been considered an alternative [32]; however, donor selection remains the greatest challenge in this setting. In addition, ethical development, standardization, and adequate stepwise preclinical and clinical evaluations are needed to advance the clinical use of allogeneic PRP, as has been the case for autologous PRP.

Other options include platelet lysates (HPLs) and platelet-derived extracellular vesicles (p-EVs) [33,34,35]. These medicinal products offered several benefits, including the use of surplus or nearly expired platelets from transfusions. Donor (or platelet concentrate) selection does not guarantee the product’s quality or potency. If young (e.g., non-adult) healthy donors can be selected for this specific use without complex ethical restrictions, and high-quality, more potent HPLs and p-EVs with minimal immunoreactivity can be easily produced, this could be a game changer in regenerative medicine using PRP therapy [36].

However, with respect to patient condition, although difficult to describe in detail, it is generally important to maintain a younger biological age than chronological age by promoting health and reducing chronic inflammation and lifestyle-related diseases. This is because PRP does not function independently but depends on the patient’s regenerative potential and on preceding or concurrent therapy.

To achieve more predictable and effective PRP therapy, this Special Issue will continue to be an active platform for data and concept exchange in the second edition.

## References

[1] Giordano S., Romeo M., di Summa P., Salval A., Lankinen P. (2018). A Meta-analysis On Evidence Of Platelet-rich Plasma for Androgenetic Alopecia. Int. J. Trichology.

[2] Grassi A., Napoli F., Romandini I., Samuelsson K., Zaffagnini S., Candrian C., Filardo G. (2018). Is Platelet-Rich Plasma (PRP) Effective in the Treatment of Acute Muscle Injuries? A Systematic Review and Meta-Analysis. Sports Med..

[3] Karjalainen T., Richards B., Buchbinder R. (2022). Platelet-rich plasma injection for tennis elbow: Did it ever work?. BMJ Open Sport Exerc. Med..

[4] Saita Y., Kobayashi Y., Uchino S., Nishio H., Wakayama T., Fukusato S., Momoi Y., Nakajima R., Yamamoto N., Hada S. (2025). Platelet-rich plasma therapy for knee osteoarthritis: Insights from real-world clinical data in Japan. Regen. Ther..

[5] Hussain N., Johal H., Bhandari M. (2017). An evidence-based evaluation on the use of platelet rich plasma in orthopedics—A review of the literature. Sicot-J.

[6] Pretorius J., Habash M., Ghobrial B., Alnajjar R., Ellanti P. (2023). Current Status and Advancements in Platelet-Rich Plasma Therapy. Cureus.

[7] Salgado-Pacheco V., Serra-Mas M., Otero-Viñas M. (2024). Platelet-rich plasma therapy for chronic cutaneous wounds stratified by etiology: A systematic review of randomized clinical trials. Drugs Ther. Perspect..

[8] Atiyeh B., Oneisi A., Ghieh F. (2021). Platelet-Rich Plasma Facial Rejuvenation: Myth or Reality?. Aesthetic Plast. Surg..

[9] Wang H.-L., Avila G. (2007). Platelet rich plasma: Myth or reality?. Eur. J. Dent..

[10] Wojtys E.M. (2012). The PRP Question. Sports Health.

[11] Raeissadat S.A., Rayegani S.M., Jafarian N., Heidari M. (2022). Autologous conditioned serum applications in the treatment of musculoskeletal diseases: A narrative review. Future Sci. OA.

[12] Nadelmann J.B., Bunya V.Y., Ying G.S., Hua P., Massaro-Giordano M. (2022). Effect of Autologous Platelet-Rich Plasma Drops in the Treatment of Ocular Surface Disease. Clin. Ophthalmol..

[13] Masuki H., Okudera T., Watanabe T., Suzuki M., Nishiyama K., Okudera H., Nakata K., Uematsu K., Su C.Y., Kawase T. (2016). Growth factor and pro-inflammatory cytokine contents in PRP, plasma rich in growth factors (PRGF), advanced-platelet-rich fibrin (A-PRF) and concentrated growth factors (CGF). Int. J. Implant. Dent..

[14] Kobayashi E., Fluckiger L., Fujioka-Kobayashi M., Sawada K., Sculean A., Schaller B., Miron R.J. (2016). Comparative release of growth factors from PRP, PRF, and advanced-PRF. Clin. Oral Investig..

[15] O’Shaughnessey K., Matuska A., Hoeppner J., Farr J., Klaassen M., Kaeding C., Lattermann C., King W., Woodell-May J. (2014). Autologous protein solution prepared from the blood of osteoarthritic patients contains an enhanced profile of anti-inflammatory cytokines and anabolic growth factors. J. Orthop. Res..

[16] Hix J., Klaassen M., Foreman R., Cullen E., Toler K., King W., Woodell-May J. (2017). An Autologous Anti-Inflammatory Protein Solution Yielded a Favorable Safety Profile and Significant Pain Relief in an Open-Label Pilot Study of Patients with Osteoarthritis. BioRes. Open Access.

[17] Berrigan W., Tao F., Kopcow J., Park A.L., Allen I., Tahir P., Reddy A., Bailowitz Z. (2024). The Effect of Platelet Dose on Outcomes after Platelet Rich Plasma Injections for Musculoskeletal Conditions: A Systematic Review and Meta-Analysis. Curr. Rev. Musculoskelet. Med..

[18] Corsini A., Perticarini L., Palermi S., Bettinsoli P., Marchini A. (2025). Re-Evaluating Platelet-Rich Plasma Dosing Strategies in Sports Medicine: The Role of the “10 Billion Platelet Dose” in Optimizing Therapeutic Outcomes-A Narrative Review. J. Clin. Med..

[19] Mittal M., Siddiqui M.R., Tran K., Reddy S.P., Malik A.B. (2014). Reactive oxygen species in inflammation and tissue injury. Antioxid. Redox Signal..

[20] Forrester S.J., Kikuchi D.S., Hernandes M.S., Xu Q., Griendling K.K. (2018). Reactive Oxygen Species in Metabolic and Inflammatory Signaling. Circ. Res..

[21] Ushiki T., Mochizuki T., Osawa M., Suzuki K., Tsujino T., Watanabe T., Mourão C.F., Kawase T. (2024). Plasma Gel Matrix as a Promising Carrier of Epigallocatechin Gallate for Regenerative Medicine. J. Funct. Biomater..

[22] Fujioka-Kobayashi M., Schaller B., Mourão C., Zhang Y., Sculean A., Miron R.J. (2021). Biological characterization of an injectable platelet-rich fibrin mixture consisting of autologous albumin gel and liquid platelet-rich fibrin (Alb-PRF). Platelets.

[23] Kargarpour Z., Nasirzade J., Panahipour L., Miron R.J., Gruber R. (2020). Liquid Platelet-Rich Fibrin and Heat-Coagulated Albumin Gel: Bioassays for TGF-β Activity. Materials.

[24] Miron R.J., Pikos M.A., Estrin N.E., Kobayashi-Fujioka M., Espinoza A.R., Basma H., Zhang Y. (2000). Extended platelet-rich fibrin. Periodontology.

[25] Deucher A.J., Simon K.A., Vinha P.P., Fiori W. (2018). P-245—PRP as an antioxidant strategy. Free Radic. Biol. Med..

[26] Carlsen M.H., Halvorsen B.L., Holte K., Bøhn S.K., Dragland S., Sampson L., Willey C., Senoo H., Umezono Y., Sanada C. (2010). The total antioxidant content of more than 3100 foods, beverages, spices, herbs and supplements used worldwide. Nutr. J..

[27] Kawase T., Mourão C.F., Fujioka-Kobayashi M., Mastrogiacomo M. (2024). Editorial: Recent advances in platelet-concentrate therapy in regenerative medicine. Front. Bioeng. Biotechnol..

[28] Ota M., Okumo T., Sato A., Nagasaka R., Mukunoki M., Izukashi K., Oike J., Yagura S., Koya T., Kanzaki K. (2024). Prognostic Factors in Intra-articular Platelet-Rich Plasma Treatment for Knee Osteoarthritis: A Comparative Analysis of Responders and Nonresponders. Cureus.

[29] Wakayama T., Saita Y., Uchino S., Kobayashi Y., Nishio H., Fukusato S., Momoi Y., Ikeda H., Kaneko K., Ishijima M. (2025). MRI-Based Evaluation of PRP Therapy in Knee Osteoarthritis: WORMS and Synovial Changes at 6 Months. J. Clin. Med..

[30] Kawase T., Okuda K. (2018). Comprehensive Quality Control of the Regenerative Therapy Using Platelet Concentrates: The Current Situation and Prospects in Japan. BioMed Res. Int..

[31] Kawase T. (2015). Platelet-rich plasma and its derivatives as promising bioactive materials for regenerative medicine: Basic principles and concepts underlying recent advances. Odontology.

[32] Akbarzadeh S., McKenzie M.B., Rahman M.M., Cleland H. (2021). Allogeneic Platelet-Rich Plasma: Is It Safe and Effective for Wound Repair?. Eur. Surg. Res..

[33] Puhm F., Boilard E., Machlus K.R. (2021). Platelet Extracellular Vesicles: Beyond the Blood. Arterioscler. Thromb. Vasc. Biol..

[34] Muttiah B., Ng S.L., Lokanathan Y., Ng M.H., Law J.X. (2024). Beyond Blood Clotting: The Many Roles of Platelet-Derived Extracellular Vesicles. Biomedicines.

[35] Burnouf T., Strengers P., Busch M.P. (2025). Beyond transfusion: Platelet-derived therapeutic products as a new frontier for blood establishments and transfusion medicine. Vox Sang..

[36] Burnouf T., Chou M.L., Lundy D.J., Chuang E.Y., Tseng C.L., Goubran H. (2023). Expanding applications of allogeneic platelets, platelet lysates, and platelet extracellular vesicles in cell therapy, regenerative medicine, and targeted drug delivery. J. Biomed. Sci..

